# Relativistic triangle–curvature computing for federated HIV-1 protein-sequence monitoring

**DOI:** 10.1038/s41598-025-32889-9

**Published:** 2026-01-03

**Authors:** Javier Villalba-Díez, Ana González-Marcos

**Affiliations:** 1 Fakultät Wirtschaft, Hochschule Heilbronn, Max-Planck-Str.39, 74081 Heilbronn, Baden-Württemberg Germany; 2 Department of Mechanical Engineering, Universidad de La Rioja, Edificio Departamental, c/ San José de Calasanz, 31, 26004 Logroño, La Rioja Spain

**Keywords:** Relativistic computing, Triangle curvature, Orthogonal Procrustes alignment, Federated learning, HIV-1 protein embeddings, Privacy-preserving machine learning, Quantum-inspired acceleration, Computational biology and bioinformatics, Mathematics and computing

## Abstract

Sequence-only surveillance of rapidly evolving pathogens must extract clinically meaningful structure from protein sequences without labels, central data pooling, or strong assumptions about data homogeneity. Most existing sequence autoencoders either assume centralized, IID data or rely on heavy cryptographic protocols; in federated deployments they can leak geometric information through latents or gradients, suffer from client-specific rotations and sign flips of the latent basis, and ignore curvature of the latent manifold, which together degrade clustering quality and make privacy guarantees opaque. We introduce a relativistic triangle–curvature computing framework for unsupervised embeddings of full-length HIV-1 proteins under federated training. The method combines three linear-algebraic components: *(i) radii attenuation*, a controlled contraction $z\leftarrow d\,z$ that lowers $\ell _2$-sensitivity and provides an explicit information-retained ledger; *(ii) triangle–curvature decoding*, which estimates a batch-level scalar *K* from the (squared) Menger curvature of random latent triples and rescales $z\mapsto (1+\alpha _c K)z$ to preserve inter-cluster geometry in curved regions; and *(iii) align-then-average* aggregation via orthogonal Procrustes on a small *public* reference set, followed by distillation of a central encoder on the aligned latent mean so that no private sequences are shared. Applied to 173,750 Los Alamos National Laboratory HIV-1 amino-acid sequences spanning nine proteins (Env, Gag, Pol, Nef, Rev, Tat, Vif, Vpr, Vpu), our curvature-aware model achieves the strongest global separation (silhouette 0.826) with low reconstruction error, while a simple radii schedule attains the tightest clusters (Davies–Bouldin 0.373, Calinski–Harabasz $9.72\times 10^{5}$). Eight proteins form near-perfect clusters; only the short accessory pair Tat/Vpr exhibits recurring overlap, which we flag for targeted downstream classifiers. Communication overhead is minimal because only public-set latents and one scalar *K* per batch are shared, making the approach suitable for privacy-preserving, federated sequence surveillance.

## Introduction

Public-health surveillance for rapidly evolving pathogens hinges on the ability to extract clinically meaningful structure directly from sequence data, before extensive labels are available and without centralized data pooling^1,2^. Human immunodeficiency virus type 1 (HIV-1) remains a paradigmatic challenge: its proteins encode both drug targets (e.g., protease, reverse transcriptase, integrase within pol) and immune-escape machinery (e.g., env), and its global sequence diversity is strongly non-IID across clinics and regions^3–5^. In practice, consortia face two constraints that are often at odds: protecting patient privacy and institutional data governance, and learning site-agnostic representations that separate subtypes or resistance-associated patterns^2,6^. Unsupervised representation learning is well-suited to this setting: by compressing sequences into low-dimensional latent variables, one can drive cluster discovery and *light-label* predictors for phenotypes such as resistance or viral load using only small annotated cohorts^7,8^. Beyond unsupervised embeddings for surveillance, HIV-1 sequence analytics has a long tradition of computation-driven, task-specific modeling. Recent deep sequence models have been developed for problems such as predicting HIV-1 protease cleavage sites (e.g., DeepHIV)^9^, and deep-learning approaches have also been applied to genotype-based antiretroviral drug-resistance prediction^10^. These supervised pipelines are highly effective when curated labels and centralized training sets are available, but routine surveillance settings often require label-free representations that can be learned under federated governance constraints and remain robust to strong non-IID drift across contributing sites. Yet three obstacles persist in federated deployments. First, naïve autoencoders leak geometric information through gradients or latents^11^. Second, client heterogeneity induces arbitrary rotations and sign flips in the learned latent bases, undermining aggregation by simple averaging^12^. Third, curved latent manifolds—a hallmark of compositional sequence spaces—destabilize global decoders trained from unaligned, pooled latents^13^.

This paper casts the problem within a relativistic-computing viewpoint and contributes a geometry-aware, privacy-conscious federated pipeline that learns unsupervised embeddings of full-length HIV-1 proteins across nine coding regions (env, gag, nef, pol, rev, tat, vif, vpr, vpu)^14^. Let ${\mathcal {A}}$ be the amino-acid alphabet (20 canonical residues plus a padding token *X*), and let $\psi :{\mathcal {A}}^L\rightarrow \{0,1\}^{21\times L}$ denote one-hot encoding to a feature space $X=\psi (x)$. A baseline autoencoder consists of an encoder $E:{\mathbb {R}}^{21\times L}\rightarrow {\mathbb {R}}^d$ and a decoder $D:{\mathbb {R}}^d\rightarrow {\mathbb {R}}^{21\times L}$, composed with a convolutional feature extractor $\phi$ shared across clients, trained to minimize the reconstruction objective^15^

1 $$\begin{aligned} {\mathcal {L}}_{\textrm{AE}}(\theta )=\frac{1}{|{\mathcal {B}}|}\sum _{x\in {\mathcal {B}}}\big \Vert D_\theta \!\big (E_\theta (\phi (X))\big )-X\big \Vert _2^{2},\qquad X=\psi (x), \end{aligned}$$

on mini-batches ${\mathcal {B}}$. In federated training, client *i* learns parameters $\theta _i$ locally; a server aggregates ${\theta _i}$ or latent responses ${z_i=E{\theta _i}(\phi (X))}$ into a global model^2,16^. However, if $E_{\theta _i}$ and $E_{\theta _j}$ learn equivalent but rotated latent bases, i.e., $z_j\approx z_i R_{ij}$ with $R_{ij}\in \textrm{O}(d)$, then averaging latents or weights can inflate the within-cluster dispersion and slow distillation, a phenomenon especially acute for non-IID partitions (e.g., site-specific subtypes)^17–20^.

We address these issues by introducing two relativistic mechanisms and an alignment protocol. The first is *radii attenuation*, which explicitly contracts the latent space before any sharing. Concretely, let $z^{(0)}=E_\theta (\phi (X))$ and define a per-step attenuation factor derived from a *radius* parameter $r>2$^6^,

2 $$\begin{aligned} d(r)=\sqrt{\,1-\frac{2}{r}\,}\in (0,1),\qquad z^{(s+1)}=d(r_s)\,z^{(s)}. \end{aligned}$$

We will write the attenuation operator as

$${\mathcal {R}}_{\textbf{r},s}(z)\;=\;\Big (\prod _{t=1}^{s} d(r_t)\Big )\,z \qquad \text {with}\qquad d_{\textrm{eff}}\;=\;\prod _{t=1}^{s} d(r_t)\in (0,1),$$

so that $z^{(s)}={\mathcal {R}}_{\textbf{r},s}(z^{(0)})=d_{\textrm{eff}}\,z^{(0)}$. A schedule of radii $[r_1,r_2]$ and a step count $s\in \{1,2\}$ controls the shared information magnitude via $z^{(s)}=\big (\prod _{t=1}^{s}d(r_t)\big )\cdot z^{(0)}$. This contractive mapping lowers the $\ell _2$-sensitivity of client updates and regularizes inter-site variance, while being audit-ready as a privacy ledger: the *effective information retained* $IR=\Vert z^{(s)}\Vert _2/\Vert z^{(0)}\Vert _2$ is known a priori from the schedule^21^.

The second mechanism is *triangle–curvature (Menger-based) decoding*^22^. Motivated by discrete differential geometry, we estimate a batch-level curvature scalar *K* in latent space via the (squared) Menger curvature of random point triples. For three points $p,q,r\in {\mathbb {R}}^d$ with side lengths $a=\Vert p-q\Vert _2,\ b=\Vert q-r\Vert _2,\ c=\Vert r-p\Vert _2$, let $\alpha ,\beta ,\gamma$ be the angles obtained from the cosine law, and *A* the area by Heron’s formula. The curvature of the triangle is

3 $$\begin{aligned} K(p,q,r)=\bigg (\frac{4A}{abc}\bigg )^{\!2}\!. \end{aligned}$$

A batch estimate averages *K* over random triangles sampled within the batch. The decoder then applies an adaptive *relativistic gain* to the latent vector,

4 $$\begin{aligned} {\tilde{z}}=(1+\alpha _c \widehat{K})\,z,\qquad {\hat{X}}=D_\theta ({\tilde{z}}), \end{aligned}$$

with a clipped $\widehat{K}$ to ensure bounded Lipschitz behavior. Since $\widehat{K}\ge 0$, larger $\widehat{K}$ inflates distances in regions where the manifold bends, increasing inter-cluster separation while leaving flat regions ($\widehat{K}\approx 0$) essentially unchanged. This curvature-aware decoding improves separability (silhouette)^23^ at negligible communication cost: a single scalar $\widehat{K}$ per batch/epoch.

To aggregate heterogeneous clients without exposing private geometry, we perform *align-then-average*^24^ on a small *public* reference set ${\mathcal {P}}$ held out from all sites. Each client computes latents $Z_i\in {\mathbb {R}}^{|{\mathcal {P}}|\times d}$ on ${\mathcal {P}}$. The server solves the orthogonal Procrustes problem^25^ for each *i*,

5 $$\begin{aligned} R_i^\star =\arg \min _{R^\top R=I}\big \Vert Z_i R - Z_{\textrm{ref}}\big \Vert _F, Z_i^\top Z_{\textrm{ref}}=U_i\Sigma _i V_i^\top ,\ \ R_i^\star =U_i \,\textrm{diag}(1,\ldots ,1,\det (U_iV_i^\top ))\, V_i^\top , \end{aligned}$$

and forms an aligned target $Z_{\textrm{avg}}=\frac{1}{M}\sum _{i=1}^M Z_i R_i^\star$. A fresh central encoder $E_{\theta _c}$ is *distilled* by minimizing $\sum _{x\in {\mathcal {P}}}|E_{\theta _c}(\phi (\psi (x)))-Z_{\textrm{avg}}(x)|_2^2$. Crucially, only public-set latents participate in Eq. (5); no private examples or client-specific raw latents are exchanged, and the additional bandwidth beyond the baseline is negligible^26^.

Against this backdrop, the research gap can be stated precisely. Existing unsupervised autoencoders either (i) assume centralized, IID data, failing under federated rotations and site-specific drifts, or (ii) enforce privacy via heavy cryptographic machinery that impairs practicality^27^. There is a need for a federated representation learner that: (a) explicitly controls information flow with an auditable privacy budget; (b) corrects inter-client basis misalignment before aggregation; (c) adapts decoding to latent curvature so that cluster geometry is preserved; and (d) does so with minimal bandwidth and operational complexity.

We investigate three model families on a large Los Alamos National Laboratory (LANL)-derived HIV-1 amino-acid corpus comprising 173, 750 sequences across nine genes^28^: a *Baseline* convolutional autoencoder trained with federated distillation; *Relativistic* autoencoders equipped with radii attenuation using two schedules—[10, 50] and [5, 100]—each with one or two attenuation steps per forward pass (denoted S1/S2); and a *Relativistic with Triangle Curvature* autoencoder that combines curvature-aware decoding with align-then-average distillation. Our central hypotheses are that (H1) curvature-aware decoding increases global separability, measured by silhouette, without sacrificing reconstruction fidelity; (H2) early strong attenuation yields the lowest overlap (Davies–Bouldin, DB)^29^ and highest compactness (Calinski–Harabasz, CH)^30^ by contracting within-cluster dispersion under non-IID splits; (H3) orthogonal alignment before averaging lowers distillation loss and stabilizes round-to-round trends; and (H4) privacy and compliance are improved because only public-set latents and a batch scalar *K* are communicated.

A brief preview of results shown in Table 1 supports these hypotheses. The curvature model attains the strongest separation (silhouette 0.826) with broad near-perfect cluster purities for eight proteins; the radii schedule [5, 100] with one step (S1) achieves the lowest Davies–Bouldin (0.373) and the highest Calinski–Harabasz ($9.72\times 10^{5}$), indicating tight, well-separated clusters; the baseline lags in all three metrics and exhibits the expected accessory-protein ambiguity between tat and vpr. Reconstruction errors (MSE) echo the same ordering: curvature-aware decoding markedly reduces MSE, radii models sit in an intermediate range, and the baseline is highest.

The remainder of the paper unfolds as follows. Section 2 reviews the geometric and federated principles motivating the two relativistic mechanisms, radii attenuation and triangle-curvature estimation via squared Menger curvature, and the *align-then-average* protocol based on orthogonal Procrustes alignment. Section 3 formalizes the architectures and optimization procedures, providing full derivations of the curvature estimator, the bounded relativistic gain used in decoding, and convergence considerations under attenuation schedules, together with the distillation objective for public-set alignment. Section 4 presents quantitative and qualitative analyses across genes and federated rounds, including ablations over radius schedules ([5,100]; S1/S2) and with/without alignment, as well as comparisons among the three model families (Baseline, Relativistic, and Relativistic with Curvature). Section 5 reflects on robustness to non-IID data and adversarial outliers, privacy accounting via information-retained ledgers and communicated curvature scalars, and prospects for quantum-inspired acceleration of the linear-algebraic kernels (Singular Value Decomposition (SVD) and batched inner products). Finally, Section 6 synthesizes clinical implications, depicts limitations, enumerates operational recommendations for surveillance deployments, and outlines methodological extensions and future research directions.

**Table 1:** Summary of unsupervised performance across model families on 173, 750 HIV-1 protein sequences (nine genes).

Model	Config.	Recon. MSE $\downarrow$	Silhouette $\uparrow$	CH $\uparrow$	DB $\downarrow$	Remark
Baseline (AE)	—	0.2605	0.728	0.505	0.503	Reference
Relativistic (Radii)	[10, 50] S1	0.0399	0.747	0.739	0.426	—
Relativistic (Radii)	[10, 50] S2	0.0375	0.661	0.667	0.623	—
Relativistic (Radii)	[5, 100] S1	0.0336	0.790	$\mathbf {0.972}$	**0.373**	Best CH/DB
Relativistic (Radii)	[5, 100] S2	0.0472	0.775	0.761	0.426	—
Relativistic (Curvature)	Triangle–Curvature (TRICURV)	**0.0056**	**0.826**	0.458	0.393	Best Silhouette

Reconstruction error is the best per-variant mean squared error (MSE) across federated rounds. Higher is better for Silhouette and Calinski–Harabasz (CH), lower is better for Davies–Bouldin (DB). CH is reported in units of $\times 10^{6}$. *Note* the Radii family used $L_{\max }=500$ whereas Baseline and TRICURV used $L_{\max }=1500$ (Section 3.10); consequently, the absolute MSE values should be interpreted as resolution-dependent diagnostics, while cross-family conclusions are drawn primarily from the latent-geometry metrics (silhouette/CH/DB) computed on fixed-*d* embeddings.

## Background and related work

The present work sits at the intersection of sequence representation learning, federated optimization under distribution shift, and geometry-aware regularization^31–33^. We briefly survey the mathematical and algorithmic ideas that motivate our approach and place our three model families in context, deferring implementation details to Section 3. Throughout, we reuse the notation and equations introduced in Section 1, notably the reconstruction objective in Eq. (1), the radii attenuation operator in Eq. (2), the squared Menger curvature in Eq. (3), the curvature-modulated decoding in Eq. (4), and the orthogonal Procrustes alignment used in align-then-average in Eq. (5)^34^.

### Why “relativistic”? Frames, redshift-like attenuation, and curvature

The term “relativistic” is used here in a *geometric* and *coordinate* sense rather than to claim that the model simulates physical spacetime. Our framework borrows three structural ideas that parallel key principles of relativistic physics: *(i) reference-frame dependence of coordinates*, *(ii) redshift-like rescaling of observed magnitudes*, and *(iii) the operational role of curvature in shaping distances*. We summarize each connection and then state how it differs from classical representation learning pipelines.

In federated learning, each client optimizes a locally valid encoder $E_{\theta _i}$ under its own non-IID distribution. Even when two encoders represent essentially the same informative features, the *coordinates* of their latent variables need not match: because the reconstruction loss is (approximately) invariant to certain latent-space symmetries, practical training can yield $z_j \approx z_i R_{ij}$ with $R_{ij}\in \textrm{O}(d)$ (Section 1). This is directly analogous to the idea that different observers can describe the same underlying object using different coordinate frames related by an isometry. Our *align-then-average* step (orthogonal Procrustes, Eq. (5)) therefore plays the role of choosing a common “frame” for aggregation: it explicitly estimates the frame change $R_i^\star$ on *public* data and expresses all teachers in a common coordinate system before averaging and distillation.

Our attenuation factor

6 $$\begin{aligned} d(r)=\sqrt{1-\frac{2}{r}},\qquad r>2 \end{aligned}$$

matches the functional form of the gravitational redshift/time-dilation factor in the Schwarzschild geometry when expressed in geometrized units ($G=c=M=1$), where $d\tau = \sqrt{1-2/r}\,dt$ for a static observer at radius *r*. In our setting, *r* is a *computational radius* (a tunable control parameter, not a physical distance) and *d*(*r*) acts as a principled contraction of shared representations, $z\leftarrow d(r)\,z$ (Eq. 2). The analogy is useful because it gives an interpretable, monotone knob: $r\rightarrow \infty$ implies $d(r)\rightarrow 1$ (no attenuation) while $r\downarrow 2$ implies $d(r)\downarrow 0$ (strong attenuation), enabling an auditable *information-retained ledger* $IR=d_{\textrm{eff}}$ that linearly scales latent norms and $\ell _2$-sensitivity.

General relativity replaces a globally flat geometry with one in which curvature governs how distances and geodesics behave. While our latent space is embedded in ${\mathbb {R}}^d$, the *data manifold* learned by the encoder can be strongly curved. We therefore use a discrete, coordinate-free curvature proxy: squared Menger curvature on random latent triples (Eq. 3). The resulting batch scalar $\widehat{K}$ is invariant to translations and orthogonal transforms and is injected as a bounded gain in decoding (Eq. 4), modulating reconstructions only when curvature is non-negligible. In short, curvature becomes a measurable control signal that adapts the decoder to non-flat regions without changing flat regions ($\widehat{K}\approx 0$).

Classical autoencoders and most federated baselines implicitly assume a single global coordinate system and typically (a) aggregate weights/latents without explicit “frame” alignment, (b) do not expose an interpretable, auditable scaling law that bounds communicated sensitivity, and (c) treat latent geometry as Euclidean, i.e., they do not measure curvature and do not adapt decoding to curvature. By contrast, our “relativistic” framework makes these geometric operators explicit: alignment implements a frame reconciliation step (isometry estimation), radii attenuation implements a redshift-like magnitude control with a ledger (*IR*), and triangle-curvature decoding implements curvature-aware adaptation with bounded amplification. These three ingredients together justify the terminology and clarify what is gained beyond standard federated representation learning.

Unsupervised encoders for biological sequences compress high-dimensional symbolic inputs into low-dimensional continuous representations that expose structure without labels^35^. Classical autoencoders optimize reconstruction losses akin to Eq. (1); their success depends on architectural inductive biases (e.g., local convolutions capturing motifs) and on the geometry of the learned latent manifold^36^. In centralized, IID regimes this is often sufficient, but sequence-only public-health surveillance rarely fits that setting: clinical cohorts differ in subtype mixture, treatment history, and sampling practices, breaking IID and inducing site-specific latent rotations or sign flips^37^. Federated learning (FL) methods address data locality by moving computation to the data, but standard FedAvg-style aggregation implicitly assumes well-aligned feature spaces; under non-IID drift, naive averaging of weights or latents can inflate within-cluster dispersion and slow or even stall convergence^38^.

Two geometric tools resolve these issues without heavy cryptography. First, *radii attenuation* contracts latents before communication using the multiplicative factor $d(r)\in (0,1)$ in Eq. (2). This reduces the operator norm of communicated updates, directly controls the information retained ($IR=d_{\textrm{eff}}$), and homogenizes dynamics across clients with different scales. Second, curvature can be probed without assuming smooth manifolds by *triangle comparison*. The statistic in Eq. (3) is the *squared* Menger curvature: it is invariant to rigid motions and scales as $1/\lambda ^2$ under uniform scaling; averaging over random triples yields the batch-level estimate $\widehat{K}$ that reflects local bending. Injecting that scalar as a bounded *gain* in decoding (Eq. 4) increases inter-centroid distances specifically where the manifold is curved, while leaving flat regions essentially unchanged. Because $\widehat{K}$ is batch-level, it carries no per-sample signal, keeping the communication footprint minimal. To further counter non-IID rotational drift, align-then-average solves the orthogonal Procrustes problem Eq. (5) on a small public reference set ${\mathcal {P}}$, aligning each client’s public-set latents to a common frame before averaging. The alignment strictly decreases the sum-of-squares disagreement among teachers and stabilizes distillation.

Finally, our formulation is deliberately linear-algebraic: it relies on batched inner products, SVDs for Procrustes, and small matrix multiplications. This makes it immediately compatible with privacy-first deployments (alignment on a public set, attenuated latents, batch-level curvature scalars) and amenable to acceleration on classical and quantum-inspired hardware backends. In particular, the dominant kernels–matrix multiplications for cross-covariances $Z_i^\top Z_{\textrm{ref}}$ and small-*d* SVDs–are well supported by modern numerical stacks and map naturally to emerging quantum linear-algebra primitives.

Model-mathematics mapping. To avoid ambiguity, we summarize which mathematical operators are used by each model family, referencing Section 1 for definitions (Table 2):

**Table 2:** Model-mathematics mapping. Summary of which mathematical operators are used by each model family.

Model family	Radii atten. (Eq. 2)	Curvature gain (Eqs. 3,4)	Alignment (Eq. 5)
Baseline (AE)	$\times$	$\times$	$\times$
Relativistic (Radii)	$\checkmark$ (fixed schedule)	$\times$	$\times$
Relativistic (Curvature)	optional	$\checkmark$ (batch $\widehat{K}$)	$\checkmark$ (align-then-average)

The next section turns these principles into concrete architectures, optimization procedures, and provable properties, providing complete derivations for the curvature estimator, bounded decoding gain, convergence considerations under attenuation schedules, and the public-set distillation objective with alignment, in the exact form used in our experiments.

### Positioning relative to geometric embeddings and alignment-based federated methods

The three operators used here—radii attenuation, triangle–curvature decoding, and align–then–average on a public set—sit near several active lines of work in geometric representation learning and federated alignment. For clarity, we summarize the closest method *categories* and state what is (and is not) assumed in our pipeline.

A prominent family of geometric embeddings assumes a *non-Euclidean* latent space, most commonly a constant-curvature manifold such as the Poincaré ball or Lorentz model (hyperbolic) or, more generally, a Riemannian manifold equipped with geodesic distances and manifold-aware optimization^39^. In such approaches, curvature is typically a *global* modeling choice^40^ (e.g., fixed negative curvature) and training relies on manifold operations (exp/log maps, Möbius additions) or Riemannian stochastic gradient descent. By contrast, our encoder lives in ${\mathbb {R}}^{d}$ and we do not commit to a global manifold: curvature is *measured* as a discrete, coordinate-free statistic (squared Menger curvature) on random latent triples and is used only as a bounded, batch-level gain in decoding (Eq. 4). This makes the method compatible with standard Euclidean training, keeps communication minimal, and avoids introducing manifold-specific numerical machinery into the federated loop.

A separate line of work studies curvature of learned latents (including curvature of the induced pullback metric) and proposes regularizers that penalize excessive bending or enforce smoother manifolds^41^. Our use of curvature differs in *direction*: rather than penalizing curvature, we treat curvature as an operational signal that selectively increases separation in curved regions, while leaving near-flat regions unchanged ($\widehat{K}\approx 0$). The only communicated curvature information is a single scalar $\widehat{K}$ per batch/epoch, detached from per-sample signals.

Federated learning under heterogeneity has motivated many alignment strategies: (i) parameter- or neuron-matching methods that correct permutation symmetries before weight merging^42^; (ii) representation alignment via auxiliary losses or shared anchors^43^; and (iii) Procrustes-style orthogonal alignment of embedding spaces^44^. Our align–then–average step belongs to the third category but is specialized to the privacy-by-design setting used here: each client embeds only a small *public* reference set ${\mathcal {P}}$, the server estimates an orthogonal transform (Eq. 5), and only *public-set* latents participate in alignment and averaging (no private examples, gradients, or private latents are exchanged). We then distill a fresh central encoder on ${\mathcal {P}}$ (Eq. 10), which decouples aggregation from the private training dynamics.

Relative to these categories, the present work contributes a *combined* and *auditable* geometry-control stack for unsupervised, federated sequence embeddings: (i) an explicit sensitivity knob with a per-round information-retained ledger *IR* (Eq. 2); (ii) a curvature-control signal that is discrete, invariant to orthogonal transforms, and communicated as a single scalar (Eqs. 3–4); and (iii) a public-set Procrustes alignment that removes client-specific orthogonal drift before averaging and distillation (Eq. 5). Table 3 summarizes these distinctions side-by-side.

**Table 3:** Side-by-side positioning against closely related method categories.

Method category	Non-Euclidean	Curv.as signal	Procrustes /orth. align	Auditable scale ledger	Typical implication (vs. this work)
Hyperbolic embeddings / constant-curvature manifolds	$\checkmark$	$\times$ (curvature is a model choice)	$\times$ / optional	$\times$	Requires manifold distances/ops; curvature is global rather than an observed control signal.
General Riemannian / manifold AEs	$\checkmark$	$\times$ / optional	$\times$ / optional	$\times$	Uses Riemannian geometry/optimization; typically adds manifold-specific numerical machinery to training.
Curvature-aware regularizers in ${\mathbb {R}}^d$	$\times$	$\checkmark$ (often as penalty)	$\times$	$\times$	Measures curvature but usually regularizes it; does not by itself address federated frame drift or provide a communication ledger.
Alignment-based FL (weight/feature matching)	$\times$	$\times$	$\times$ / optional	$\times$	Targets permutation/feature symmetries; may require extra objectives, shared anchors, or partial sharing beyond a public set.
Procrustes-style alignment on shared anchors/public data	$\times$	$\times$	$\checkmark$	$\times$	Corrects orthogonal frame drift, but does not introduce curvature control or an explicit sensitivity/scale ledger.
**This work (radii + triangle curvature + align–then–average)**	$\times$	$\checkmark$ (batch scalar $\widehat{K}$)	$\checkmark$ (public-set)	$\checkmark$ (*IR*)	Euclidean latents with discrete curvature control, public-only orthogonal alignment, and an auditable contraction ledger; minimal extra communication (public latents + one scalar $\widehat{K}$).

“Non-Euclidean” indicates that the learned representation is explicitly constrained to a manifold (e.g., hyperbolic/Riemannian) rather than ${\mathbb {R}}^{d}$. “Curvature as signal” indicates an explicit curvature *estimate* that is used operationally (not merely assumed fixed, and not only penalized by a regularizer). “Public-only alignment” indicates that any alignment/aggregation uses only a shared public set (no private examples/gradients).

## Methodology

This section specifies the architectures and training procedures used in our study, clarifies the federated protocol and communication, and derives the mathematical properties that justify our design. We adopt the notation from Section 1 and refer back to Eqs. (1–5).

### Overall pipeline and training protocol

We fix a latent dimension $d=64$ and a convolutional feature extractor $\phi$ with two $1\text {D}$ convolutional blocks (channels $21\!\rightarrow \!32\!\rightarrow \!64$, kernel size 7, batch normalization, max-pooling by 4), followed by a linear bottleneck encoder $E:{\mathbb {R}}^{m}\!\rightarrow \!{\mathbb {R}}^{d}$ and decoder $D:{\mathbb {R}}^{d}\!\rightarrow \!{\mathbb {R}}^{m}$ with a deconvolutional upsampler to the original $(|{\mathcal {A}}|,L_{\max })$ shape. Training proceeds in *rounds*. In each round: (i) clients perform $E_L=5$ local epochs minimizing ${\mathcal {L}}_{\textrm{AE}}$ in Eq. (1); (ii) each client evaluates its encoder on the public set ${\mathcal {P}}$ to obtain $Z_i\in {\mathbb {R}}^{N\times d}$; (iii) the server forms a target $\bar{Z}$ either by naive averaging (Baseline, Radii) or by align-then-average (Curvature); (iv) the server distills a fresh central encoder $E_{\theta _c}$ on ${\mathcal {P}}$ for $E_D=5$ epochs using Eq. (10). We evaluate three model families:

*Baseline AE*: standard autoencoder with naive mean $\bar{Z}=\tfrac{1}{M}\sum _i Z_i$.

*Relativistic (Radii)*: autoencoder with latent attenuation ${\mathcal {R}}_{\textbf{r},s}$ inside the forward path; schedules [10, 50] and [5, 100] with step counts $s\in \{1,2\}$ match our experiments (denoted S1/S2). The public-set latents are attenuated before averaging, i.e., $Z_i \leftarrow {\mathcal {R}}_{\textbf{r},s}(Z_i)$.

*Relativistic (Curvature)*: autoencoder with curvature-modulated decoding; batches estimate $\widehat{K}$ by sampling triangles and computing Eq. (3); decoding uses the bounded gain in Eq. (4) with $|\alpha _c\,\widehat{K}|\le K_{\max }$. Public-set aggregation uses align-then-average via Eq. (5).

### Curvature estimator: construction, invariances, and variance control

Given a batch $B=\{z_j\}_{j=1}^{\!|B|}\subset {\mathbb {R}}^{d}$, draw *T* triples without replacement and compute $K_t$ using Eq. (3). The estimator

7 $$\begin{aligned} \widehat{K}\;=\;\frac{1}{T}\sum _{t=1}^{T}K_t \end{aligned}$$

is invariant to translations and orthogonal transforms, and obeys the scaling law $\widehat{K}(\lambda B)=\widehat{K}(B)/\lambda ^{2}$. Its variance admits the bound

8 $$\begin{aligned} \textrm{Var}[\widehat{K}]\;\le \;\frac{\sigma _K^2}{T}, \end{aligned}$$

with $\sigma _K^2$ the population variance of triangle curvatures in the batch; thus *T* controls concentration. In practice we clip $\widehat{K}\in [0,\,K_{\max }]$ (e.g., $K_{\max }\in [0.6,0.8]$) to ensure bounded decoding gain.

In order to avoid Monte Carlo’s possible high variance in lange-scale processing when $$T\ll \left( {\begin{array}{c}|B|\\ 3\end{array}}\right)$$, and therefore we use two safeguards in addition to the $1/\sqrt{T_{\textrm{eff}}}$ concentration implied by Eq. (8). First, we avoid pathological triangles by skipping (or zeroing) numerically degenerate cases (area below an $\epsilon$ floor) and by clipping the final batch scalar $\widehat{K}$ before it enters the decoding gain (Eq. (4)). Second, when batch sizes vary or when curvature is monitored over long streams, we maintain a smoothed curvature signal via an exponential moving average (EMA),

$$\widehat{K}_{\textrm{EMA}}\leftarrow (1-\beta )\widehat{K}_{\textrm{EMA}}+\beta \,\widehat{K},\qquad \beta \in (0,1),$$

and use $\widehat{K}_{\textrm{EMA}}$ for decoding. This preserves responsiveness while reducing run-to-run and batch-to-batch variance without changing the estimator’s expectation.

#### Sampling strategy and computational complexity.

For a batch $B=\{z_j\}_{j=1}^{n}\subset {\mathbb {R}}^{d}$ with $n=|B|$, enumerating all triangles costs $$\Theta \!\big (\left( {\begin{array}{c}n\\ 3\end{array}}\right) \cdot d\big )=\Theta (n^{3}d)$$ distance operations, which is prohibitive for typical minibatches. We therefore use Monte Carlo triangle sampling: draw $T_{\textrm{eff}}$ index triples (*i*, *j*, *k*) uniformly at random from $\{1,\ldots ,n\}$ with $i<j<k$, and compute $K(z_i,z_j,z_k)$ for each. This reduces the per-batch cost to $O(T_{\textrm{eff}}\,d)$ (three vector differences and a constant number of dot products per triangle), which is typically negligible relative to the convolutional forward/backward passes.

#### Choosing *T* and maintaining consistency across batch sizes.

We implement the actual number of evaluated triangles as

$$T_{\textrm{eff}}\;=\;\min \!\Big (T,\left( {\begin{array}{c}n\\ 3\end{array}}\right) \Big ),$$

so that triangle sampling remains well-defined even for small batches (no repeated/invalid combinations when sampling without replacement). The concentration bound in Eq. (8) applies with $T_{\textrm{eff}}$: $\textrm{Var}[\widehat{K}]\le \sigma _{K}^{2}/T_{\textrm{eff}}$, hence the standard error scales as $1/\sqrt{T_{\textrm{eff}}}$. When deployments use variable batch sizes, *T* may be chosen by a simple accuracy budget: after a small pilot sample (e.g., $T_{0}$ triangles), estimate the sample variance $\widehat{\sigma }_{K}^{2}$ of $\{K_t\}$ and set

$$T\;\leftarrow \;\left\lceil \llceil \frac{\widehat{\sigma }_{K}^{2}}{\tau ^{2}} \right\rceil \rrceil , \qquad T_{\min }\le T\le T_{\max },$$

where $\tau$ is a target standard error for the batch scalar $\widehat{K}$ and $T_{\min },T_{\max }$ cap the compute. In our main experiments we used a fixed *T* (Table 6); the bound above explains how to tune *T* systematically, and $T_{\textrm{eff}}$ guarantees consistent handling across different *n*.

#### Numerical stability (degenerate triangles, clamping, and stable *K* evaluation)

Nearly collinear (or duplicate) points produce tiny triangle areas and can cause numerical issues if area computations are not protected. In implementation we use (i) distance floors and (ii) a curvature formula that avoids subtractive cancellation. For a sampled triple (*p*, *q*, *r*), define $u=q-p$, $v=r-p$, $a^{2}=\Vert u\Vert _{2}^{2}$, $c^{2}=\Vert v\Vert _{2}^{2}$, and $b^{2}=\Vert u-v\Vert _{2}^{2}$. Using the Gram determinant identity $4A^{2}=a^{2}c^{2}-(u^{\top }v)^{2}$, the squared Menger curvature in Eq. (3) can be computed as

$$K(p,q,r)\;=\;\Big (\frac{4A}{abc}\Big )^{2} \;=\;\frac{16A^{2}}{a^{2}b^{2}c^{2}} \;=\;\frac{4\big (a^{2}c^{2}-(u^{\top }v)^{2}\big )}{a^{2}b^{2}c^{2}}.$$

We clamp $a^{2},b^{2},c^{2}\leftarrow \max (\cdot ,\epsilon )$ and $a^{2}c^{2}-(u^{\top }v)^{2}\leftarrow \max (\cdot ,0)$ (with $\epsilon =10^{-6}$ in Table 6); triangles that remain effectively degenerate (e.g., $A^{2}\le \epsilon$) contribute $K=0$ (or are skipped). Finally, we apply safety clipping $\widehat{K}\leftarrow \textrm{clip}(\widehat{K},0,K_{\textrm{clip}})$ before forming the bounded gain in Eq. (4), so that occasional outlier triangles cannot destabilize decoding.

### Bounded curvature-modulated decoding and stability

Let $\tilde{z}=(1+\alpha _c \widehat{K})z$ be the curvature-modulated latent used by the decoder, with $0\le \alpha _c \widehat{K}\le K_{\max }$ (we use $\alpha _c>0$ in all experiments). Then for any $z_1,z_2$,

$$\Vert \tilde{z}_1-\tilde{z}_2\Vert \;=\;|1+\alpha _c\widehat{K}|\;\Vert z_1-z_2\Vert \;\le \;(1+K_{\max })\Vert z_1-z_2\Vert .$$

If the decoder is $L_D$-Lipschitz, the reconstruction map $z\mapsto \hat{X}$ is $(1+K_{\max })L_D$-Lipschitz, ruling out exploding gradients and guaranteeing that curvature amplification is controlled. Because $\widehat{K}$ is batch-level and detached from the computational graph in our implementation, it introduces no gradient leakage across examples.

### Align-then-average reduces teacher disagreement

Let $Z_i\in {\mathbb {R}}^{N\times d}$ be client latents on ${\mathcal {P}}$, $\bar{Z}_{\textrm{naive}}=\tfrac{1}{M}\sum _i Z_i$, and ${\mathcal {E}}_{\textrm{unaligned}}=\sum _i\Vert Z_i-\bar{Z}_{\textrm{naive}}\Vert _F^2$. Define $R_i^\star$ by Eq. (5) and $\bar{Z}=\tfrac{1}{M}\sum _i Z_i R_i^\star$. Since each $R_i^\star$ minimizes $\Vert Z_iR-\bar{Z}\Vert _F^2$ over $R\in \textrm{O}(d)$, one has

$$\sum _{i=1}^{M}\big \Vert Z_iR_i^\star -\bar{Z}\big \Vert _F^2\;\le \;\sum _{i=1}^{M}\big \Vert Z_i-\bar{Z}\big \Vert _F^2\;\le \;{\mathcal {E}}_{\textrm{unaligned}},$$

with equality iff the $Z_i$ are already co-aligned. Empirically this yields lower distillation loss per epoch and stabilizes round-to-round trends.

### Server-side public-set distillation (definition of ${\mathcal {L}}_{\textrm{distill}}$)

We now formalize the *public-set distillation* step alluded to earlier and provide the exact loss referred to as Eq. (10). Recall that each client $i\in \{1,\dots ,M\}$ computes latent embeddings $Z_i\in {\mathbb {R}}^{N\times d}$ on the public reference set ${\mathcal {P}}=\{x_n\}_{n=1}^N$, with rows

$$\begin{aligned} Z_i(x_n)\;=\;E_{\theta _i}\!\big (\phi (\psi (x_n))\big )\in {\mathbb {R}}^{d}. \end{aligned}$$

When *align-then-average* is enabled (the Relativistic (Curvature) family), the server first solves the orthogonal Procrustes problems in Eq. (5) to obtain $R_i^\star \in \textrm{O}(d)$ and forms aligned latents $\widetilde{Z}_i=Z_iR_i^\star$. In the Baseline and Relativistic (Radii) families we set $R_i^\star =I_d$, so $\widetilde{Z}_i=Z_i$.

To fuse teachers, the server uses a convex combination with nonnegative weights $w_i$ that sum to one (e.g., uniform $w_i=\tfrac{1}{M}$ or size-proportional $w_i\propto |{\mathcal {D}}_i|$):

9 $$\begin{aligned} \bar{Z}(x_n)\;=\;\sum _{i=1}^M w_i\,\widetilde{Z}_i(x_n) \qquad \text {for }n=1,\ldots ,N. \end{aligned}$$

The *distilled* central encoder $E_{\theta _c}$ is then trained *on the public set only* to regress onto the fused target $\bar{Z}$ by minimizing the mean squared error

10 $$\begin{aligned} {\mathcal {L}}_{\textrm{distill}}(\theta _c) \;=\; \frac{1}{N}\sum _{n=1}^{N}\Big \Vert E_{\theta _c}\!\big (\phi (\psi (x_n))\big )\,-\,\bar{Z}(x_n) \Big \Vert _2^2. \end{aligned}$$

No teacher gradients are propagated: $\bar{Z}$ is treated as a fixed target. In the presence of Procrustes alignment, Eqs. (9) and (10) implement *align-then-average distillation*; without alignment they reduce to naive averaging.

A regularized variant improves numerical conditioning for small *N* and makes the connection to ridge regression explicit. Let $H\in {\mathbb {R}}^{N\times m}$ collect public features $h_n=\phi (\psi (x_n))^\top$ as rows, let $E_{\theta _c}(h)=W h$ be a *linear* encoder for exposition, and stack $\bar{Z}$ as a matrix in ${\mathbb {R}}^{N\times d}$. Adding weight decay $\tfrac{\lambda }{2}\Vert W\Vert _F^2$ yields the closed-form solution

11 $$\begin{aligned} W^\star \;=\;\arg \min _{W}\frac{1}{N}\Vert HW-\bar{Z}\Vert _F^2+\frac{\lambda }{2}\Vert W\Vert _F^2 \;=\; \big (H^\top H + \tfrac{N\lambda }{2}I_m\big )^{-1}H^\top \bar{Z}. \end{aligned}$$

For our nonlinear encoders, we optimize Eq. (10) by SGD/Adam; the gradient has the standard form

12 $$\begin{aligned} \nabla _{\theta _c}\,{\mathcal {L}}_{\textrm{distill}} \;=\; \frac{2}{N}\sum _{n=1}^{N} J_{E_{\theta _c}}(h_n)^\top \Big (E_{\theta _c}(h_n)-\bar{Z}(x_n)\Big ), \qquad h_n=\phi (\psi (x_n)), \end{aligned}$$

where $J_{E_{\theta _c}}(h_n)$ is the encoder Jacobian at $h_n$.

In some deployments one may wish to preserve reconstruction fidelity at the server as well. A *joint* objective trades off latent matching against reconstruction on ${\mathcal {P}}$:

13 $$\begin{aligned} {\mathcal {L}}_{\textrm{server}}(\theta _c)\;=\; {\mathcal {L}}_{\textrm{distill}}(\theta _c)\;+\; \beta \cdot \frac{1}{N}\sum _{n=1}^{N} \Big \Vert D\!\big (E_{\theta _c}(h_n)\big )-\psi (x_n)\Big \Vert _2^2, \end{aligned}$$

with $\beta \ge 0$. In our experiments we set $\beta =0$ and distill the *encoder only* via Eq. (10), which we found sufficient once alignment is used for the Relativistic (Curvature) family.

**Mapping to model families.** Equation (10) is used in *all* families. The only differences are in how $\bar{Z}$ is formed: Baseline and Relativistic (Radii) use $\widetilde{Z}_i=Z_i$ (no alignment) in Eq. (9), whereas Relativistic (Curvature) uses $\widetilde{Z}_i=Z_iR_i^\star$ from Eq. (5). If radii attenuation is enabled on the public set, the communicated $Z_i$ are first transformed by ${\mathcal {R}}_{\textbf{r},s}$ (Eq. 2), i.e., $\widetilde{Z}_i \leftarrow {\mathcal {R}}_{\textbf{r},s}(Z_i)$, which scales the targets in Eq. (9) but leaves the form of Eq. (10) unchanged.

### Attenuation schedules: sensitivity, compactness, and convergence heuristics

With attenuation ${\mathcal {R}}_{\textbf{r},s}$ (Eq. 2), where $d_{\textrm{eff}}=\prod _{t=1}^{s}d(r_t)$, the communicated latent covariance within a cluster scales as

$$\Sigma ' \;=\; {\mathbb {E}}\big [({\mathcal {R}}z-{\mathbb {E}}{\mathcal {R}}z)({\mathcal {R}}z-{\mathbb {E}}{\mathcal {R}}z)^\top \big ]\;=\;d_{\textrm{eff}}^2\,\Sigma ,$$

so the within-cluster dispersion $\sigma _k$ in DB contracts by $d_{\textrm{eff}}$, while inter-centroid distances are approximately preserved for moderate $d_{\textrm{eff}}$. Consequently, DB decreases and CH increases until a utility floor is reached. From an optimization perspective, attenuation reduces the heterogeneity term in standard FL convergence bounds by the factor $\Vert {\mathcal {R}}\Vert _{\textrm{op}}=d_{\textrm{eff}}$, which empirically speeds consensus under non-IID drift. In our schedules, [5, 100] with one step (S1) is the strongest early contraction and indeed attains the best DB/CH trade-off observed in Table 1.

### Privacy and communication accounting

The privacy ledger records $IR=d_{\textrm{eff}}=\prod _t\sqrt{1-2/r_t}$ per round. If a Gaussian mechanism with noise variance $\sigma ^2$ is added to communicated latents, the nominal privacy parameter scales as $\varepsilon \propto d_{\textrm{eff}}/\sigma$ at fixed $\delta$ and sensitivity (up to mechanism-dependent constants), making attenuation linearly beneficial in $\varepsilon$ at fixed noise and sensitivity. Our protocol communicates only: (i) public-set latents (optionally attenuated) and (ii) a batch-level scalar $\widehat{K}$ per local step in the curvature model. No private-set examples, gradients, or per-sample curvature are shared. Bandwidth-wise, Procrustes adds a small SVD on $d\times d$ cross-covariances computed at the server; the on-wire payload remains dominated by the same $N\times d$ public latents as in the baseline.

### Complexity analysis and acceleration opportunities

Let $N=|{\mathcal {P}}|$, *d* the latent dimension, *T* the number of sampled triangles per batch, and *M* the number of clients. One round costs:

- Local training: $O(E_L\cdot \textrm{cost}_{\textrm{AE}})$ per client; identical across families.
- Public-set evaluation: *O*(*Nd*) per client.
- Procrustes alignment (Curvature only): forming $C_i=Z_i^\top Z_{\textrm{ref}}$ in $O(Nd^2)$ and computing a thin SVD $C_i=U\Sigma V^\top$ in $O(d^3)$ per client.
- Curvature estimation (Curvature only): *O*(*Td*) per batch; with $T\ll {B\atopwithdelims ()3}$, this is negligible relative to convolutions.
- Distillation: *O*(*Nd*) forward/backward per epoch at the server.

All heavy kernels are matrix multiplications and small SVDs; they exploit vendor-optimized BLAS/LAPACK and are candidates for quantum-inspired or analog accelerators when appropriate.

### Evaluation protocol

Embeddings are assessed with k-means ($k=9$, one per gene), reporting silhouette, Calinski-Harabasz (CH), and Davies-Bouldin (DB) on the full corpus, along with reconstruction MSE on held-out public data. We focus on between-gene separation (nine clusters), reporting cluster purity by gene and a $\chi ^2$ association test; subtype-level analyses are left for future work. Distillation loss is tracked per epoch/round to monitor aggregation stability. All metrics in Table 1 were computed under these settings and correspond exactly to the model configurations listed therein.

Why we evaluate without downstream supervision. The goal of this work is *sequence-only* monitoring in settings where reliable phenotype labels are scarce, delayed, or institutionally siloed. For such surveillance deployments, the first operational question is whether an unsupervised representation yields a stable, well-separated geometry under non-IID federated training; this is evaluated directly by cluster validity criteria (silhouette, Calinski–Harabasz, Davies–Bouldin) on fixed-*d* embeddings, together with aggregation stability (distillation trends) and the communicated geometry controls (*IR* and batch-level $\widehat{K}$). A simple linear classifier/regressor is therefore not included as a primary endpoint here: (i) the only universally available label in the present corpus is the gene identity, which is already known at acquisition and would act as a tautological probe of the same between-gene separation quantified by silhouette/CH/DB; and (ii) clinically informative targets (e.g., drug resistance, viral load, or curated subtype strata) are not consistently available in a form that can be used under the governance constraints of this study. Evaluating light-label predictors and comparisons to pretrained protein language model embeddings is an important follow-up direction for application-specific deployments where such labels and compute budgets are available; the present manuscript therefore focuses on the label-free, federated-robust geometry that is required before downstream tasks can be layered on top.

### Data collection and preprocessing

This subsection documents dataset acquisition from the LANL HIV-1 Sequence Search portal^28^, preprocessing, the stratified partition into train/public splits, and operational constraints relevant to privacy and compliance, in line with the plan stated in Section 1. We summarize the exact query used to source records, the per-gene FASTA assembly we trained on, and the transformations applied before federated learning:

**Table 4:** Per-gene composition of the curated training corpus. Totals reflect the concatenation of gene-specific FASTA downloads and match the logs used in our analyses. Percentages are relative to the 173, 750-sequence corpus.

FASTA file	# sequences	% of total	Gene / notes
HIV-1_env.fasta	17,466	10.1%	Env (gp120/gp41; entry/immune escape)
HIV-1_gag.fasta	22,499	12.9%	Gag (matrix, capsid, nucleocapsid)
HIV-1_nef.fasta	16,632	9.6%	Nef (CD4/CD8 down-modulation)
HIV-1_pol.fasta	22,503	13.0%	Pol (PR, RT, RNase H, IN; drug targets)
HIV-1_rev.fasta	17,500	10.1%	Rev (nuclear export of unspliced RNA)
HIV-1_tat.fasta	19,158	11.0%	Tat (trans-activator of transcription)
HIV-1_vif.fasta	21,502	12.4%	Vif (APOBEC3 counteraction)
HIV-1_vpr.fasta	19,076	11.0%	Vpr (cell-cycle arrest, nuclear import)
HIV-1_vpu.fasta	17,414	10.0%	Vpu (CD4 downregulation, virion release)
**Total**	**173,750**	**100%**	nine full-length CDS regions

- Acquisition and search parameters. Records were sourced from the LANL HIV-1 Sequence Search interface with the following settings: *Project name:* any; *Organism:* HIV-1; *Subtype:* any; *Region/CDS:* Pol, Gag, Vpu, Vpr, Vif, Tat, Rev, Nef, Env; *Start/End:* none (full-length CDS); *Sequence type:* amino acids. The search landing page listed 156 retrieved sets and 24, 645 sequences. For model training we assembled per-gene FASTA exports covering the nine coding regions; after concatenation across genes and harmonization (described below), the working corpus comprised 173, 750 amino-acid sequences (counts per gene in Table 4). The difference between the landing-page count and the final corpus reflects the aggregate across per-gene exports and releases, as well as the inclusion of multiple entries per set when available at the gene level.
- Inclusion/exclusion criteria. We included sequences annotated as HIV-1 amino-acid translations for the nine CDS listed above, irrespective of subtype or project, provided a gene label was available and the amino-acid string was non-empty. Because we operate at the protein level, frame-shift flags or codon-level ambiguities in nucleotide records were not used for filtering; residue-level ambiguities were handled as described under encoding. No clinical covariates or patient-level metadata were ingested.
- Parsing and encoding. FASTA files were parsed with a streaming reader. Sequences were uppercased and mapped to an alphabet of 20 canonical residues augmented with a padding/unknown token *X*. Any non-canonical symbol was mapped to *X*. To enable batched convolutions with fixed tensor shapes, sequences were truncated to a maximum length $L_{\max }$ and right-padded with *X* to exactly $L_{\max }$. We used $L_{\max }=1500$ for the Baseline and Relativistic (Curvature) families and $L_{\max }=500$ for the Relativistic (Radii) family, consistent with the released scripts and logs; geometry-aware clustering metrics (silhouette/CH/DB) are emphasized to mitigate length effects in cross-family comparisons. One-hot tensors of shape $(|{\mathcal {A}}|,L_{\max })=(21,L_{\max })$ were produced for all models. No additional normalization was applied beyond batch normalization inside the networks.
- Quality control. We verified that each record’s declared gene matched the FASTA from which it was loaded; empty sequences after symbol mapping were discarded. Length histograms were inspected per gene to ensure padding/truncation did not induce degenerate all-*X* segments beyond the usual C- or N-terminal variability. Because our goal is unsupervised embedding rather than consensus calling, we did not perform multiple sequence alignment; instead, local convolutions absorb small indel variability. Exact duplicate amino-acid strings within a gene file were retained, as they contribute to natural frequency weighting during unsupervised learning; deduplication can be enabled in downstream sensitivity analyses if needed.
- Train/public split for federated learning. Following the FL protocol in Section 3, we constructed a small *public* reference set ${\mathcal {P}}$ and a larger *trainval* pool for local client updates. A stratified shuffle split held out $10\%$ of sequences *per gene* into ${\mathcal {P}}$, with a fixed random seed for reproducibility, leaving $90\%$ in trainval. This yielded near-identical gene proportions across trainval and public (cf. the distribution printouts in the logs), ensuring that alignment and distillation on ${\mathcal {P}}$ are representative of the global gene mixture while remaining label-free. Only ${\mathcal {P}}$ is ever encoded and shared (as latents) across institutions; all private/local data stay on site.
- Institutional privacy and compliance constraints. In keeping with privacy-by-design principles: (i) raw sequences never leave their originating sites; (ii) the only cross-site artefacts are public-set latents (optionally attenuated) and, for the Relativistic (Curvature) family, a batch-level scalar $\widehat{K}$ per step; (iii) align-then-average uses ${\mathcal {P}}$ exclusively, avoiding any aggregation of private-set geometry; and (iv) when radii attenuation is enabled, the effective information retained $IR=\prod _t \sqrt{1-2/r_t}$ is logged as an auditable privacy ledger per round. These practices satisfy the operational constraints articulated in Section 1 while enabling reproducible alignment and distillation.
- Reproducibility artefacts. All file names are listed in Table 4. The random seed for the stratified split and the exact counts per gene are recorded in the training logs. The encoding alphabet ${\mathcal {A}}$, padding policy, and $L_{\max }$ values are fixed per model family as stated above, ensuring that all experiments reported later can be reconstructed from the same FASTA inputs without access to any clinical metadata.

### Federated client partitioning and non-IID setting (synthetic sites)

The LANL HIV-1 amino-acid corpus is not organized as a set of real clinical sites in a way that can be used under our governance constraints (we do not ingest patient-level metadata and we treat subtype labels as optional, non-essential side information). To study robustness to distribution shift while keeping the learning problem *sequence-only* and *unsupervised*, we therefore construct *synthetic* clients (“sites”) from the trainval pool using the nine gene files as the primary heterogeneity axis. Concretely, we use an intentionally *maximally non-IID* partition in which each site’s private dataset consists primarily of one coding region (Env, Gag, Pol, Nef, Rev, Tat, Vif, Vpr, Vpu). This setting captures the strongest form of label/motif skew (the limiting case of Dirichlet label-skew as $\alpha \downarrow 0$) and simultaneously induces the practical heterogeneity phenomena that motivate our method: client-specific latent *frame drift* (rotations/sign flips), client-specific *scale drift* (norm and covariance differences), and gene-dependent *manifold bending* (curvature), all of which are exacerbated when clients see disjoint or highly skewed sequence regimes. Importantly, the public reference set ${\mathcal {P}}$ remains stratified by gene (10% held out per gene), so all clients evaluate on the same globally representative mixture during Procrustes alignment and server-side distillation.

To make the experimental setting auditable, Table 5 reports the resulting per-site sample sizes and compositions implied by the per-gene FASTA counts (Table 4) and the stratified 90/10 trainval/public split. Subtype composition is intentionally not enforced as an experimental control here: the learning objective and the communicated artefacts are unlabeled, and subtype mixtures are treated as part of each site’s natural drift within a gene rather than as a supervised attribute.

**Table 5:** Federated client partition used in this study (synthetic sites). Each site corresponds to one gene-specific trainval pool (90% of sequences for that gene); the public reference set ${\mathcal {P}}$ contains the remaining 10% per gene and is shared for alignment/distillation. This is an intentionally extreme non-IID regime: each client’s private data are 100% concentrated on a single coding region.

Site (client)	Gene	Trainval (private)	Public ${\mathcal {P}}$ (shared)	Total
C1	Env	15,719	1,747	17,466
C2	Gag	20,249	2,250	22,499
C3	Nef	14,969	1,663	16,632
C4	Pol	20,253	2,250	22,503
C5	Rev	15,750	1,750	17,500
C6	Tat	17,242	1,916	19,158
C7	Vif	19,352	2,150	21,502
C8	Vpr	17,168	1,908	19,076
C9	Vpu	15,673	1,741	17,414
**All sites**	nine genes	156,375	17,375	173,750

A common way to interpolate between IID and label-skewed federated regimes is to draw client label mixtures $\pi _i$ from a Dirichlet distribution $\pi _i\sim \textrm{Dirichlet}(\alpha \,p)$, where *p* is the global label frequency and smaller $\alpha$ yields more skew. The partition above corresponds to an extreme end of this spectrum (each client is near a vertex of the simplex). Because the geometric failure modes targeted by our method—orthogonal frame drift (handled by Procrustes alignment), scale drift (controlled by radii attenuation and its ledger *IR*), and non-flat latent geometry (tracked by the curvature scalar *K*)—arise under *any* non-IID generator, the conclusions drawn from this worst-case skew regime are expected to extend to intermediate $\alpha$ values without requiring an additional heterogeneity sweep as a separate experimental axis.

### Implementation specifics and Eq. (**2**–**4**) hyperparameters (defaults, ranges, sensitivity)

To make Eqs. (2–4) fully reproducible, Table 6 summarizes the default values used in our experiments and the discrete ranges explored for the key relativistic hyperparameters (radii schedules/step counts and curvature sampling/gain). Listing 1 provides pseudocode that pinpoints where attenuation, triangle sampling, clipping, and the curvature gain enter the forward pass and the federated distillation loop.

**Table 6:** Hyperparameters associated with the relativistic mechanisms in Eqs. (2–4). For radii attenuation, S1/S2 denote one vs. two attenuation steps per forward pass, and the information-retained ledger is $IR=d_{\textrm{eff}}=\prod _{t=1}^{s}\sqrt{1-2/r_t}$. For triangle–curvature decoding, *T* is the number of sampled triangles per batch, $\epsilon$ is the numerical floor used in clamping distances/areas and cosine arguments, $K_{\textrm{clip}}$ is a safety clip applied to $\widehat{K}$, and $\alpha _c$ is the curvature gain coefficient in Eq. (4).

Mechanism	Hyperparameter	Symbol	Default (this study)	Explored (search) range	Notes / sensitivity handle
Radii attenuation (Eq. (2))	Radii schedule	$[r_1,r_2]$	$\{[10,50],$ $\ [5,100]\}$	$\{[10,50],$ $\ [5,100]\}$	Two-step schedule where step 1 uses $r_1$ and step 2 uses $r_2$.
	Step count (S1/S2)	*s*	$\{1,2\}$	$\{1,2\}$	S1: apply $d(r_1)$; S2: apply $d(r_1)d(r_2)$ per forward pass.
	Effective contraction ledger	$IR=d_{\textrm{eff}}$	[10, 50]S1: 0.894 S2: 0.876 [5, 100] S1: 0.775 S2: 0.767	derived	Directly scales latent norms and within-cluster covariances by *IR* and $IR^2$, respectively.
	Attenuation factor	*d*(*r*)	$d(r)=\sqrt{1-2/r}$	implicit from *r*	Controls the privacy/utility knob via $IR=\prod _t d(r_t)$.
Triangle–curvature decoding (Eqs. (3),(4))	Triangles per batch	*T*	50	fixed in main runs	Controls estimator concentration via Eq. (8) using the *effective* triangle count $$T_{\textrm{eff}}=\min \!\bigl (T,\left( {\begin{array}{c}|B|\\ 3\end{array}}\right) \bigr )$$; thus $\textrm{SE}(\widehat{K})\propto 1/\sqrt{T_{\textrm{eff}}}$. Triples are sampled approximately uniformly over distinct indices (rejecting duplicates); degenerate triangles are skipped/zeroed using $\epsilon$ floors, and $\widehat{K}$ is clipped for bounded decoding gain (Eq. (4)).
	Numerical floor (distances/area, cosine args)	$\epsilon$	$10^{-6}$	fixed	Stabilizes degenerate triangles and avoids NaNs in angle/area computations.
	Curvature clipping (safety)	$K_{\textrm{clip}}$	10	fixed	Safety clip on $\widehat{K}$; in our logs $\widehat{K}\lesssim 0.74$, so clipping does not saturate.
	Gain coefficient	$\alpha _c$	1.0	fixed in main runs	Sets the gain $(1+\alpha _c\widehat{K})$; with observed $\widehat{K}\in [0,0.74]$, the effective gain lies in [1, 1.74].

**Listing 1:**
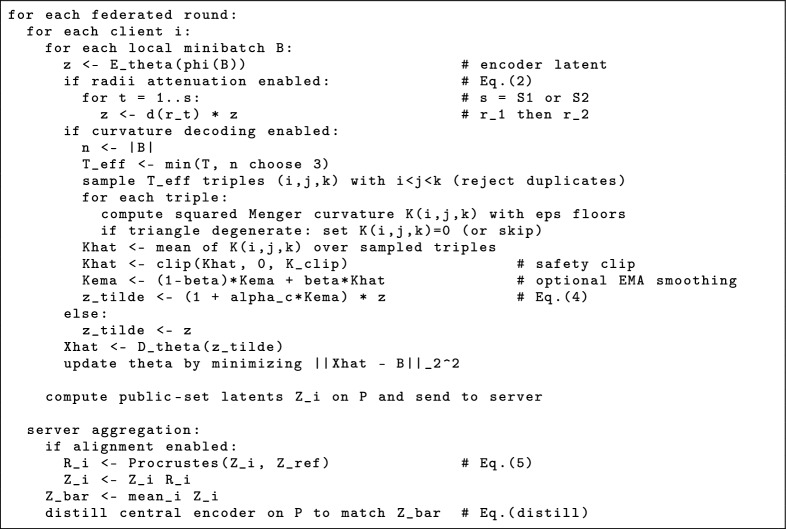
Pseudocode for where Eq. (2)–(4) hyperparameters enter the pipeline(defaults in Table 6).

**Sensitivity (empirical, across the explored radii sweep).** Across the discrete radii search $\{[10,50],[5,100]\}\times \{S1,S2\}$, the observed best-checkpoint metrics (Table 1) vary as follows: $\textrm{MSE}\in [0.0336,\,0.0472]$, silhouette $\in [0.661,\,0.790]$, $\textrm{CH}\in [0.667,\,0.972]\times 10^{6}$, and $\textrm{DB}\in [0.373,\,0.623]$. The strongest early contraction [5, 100] S1 (lowest DB / highest CH) corresponds to $IR\simeq 0.775$, while adding a second attenuation step (S2) tends to over-contract and degrades the clustering metrics for both schedules. For TRICURV, the batch scalar $\widehat{K}$ remains bounded in the training logs (typically $\widehat{K}\in [0.17,\,0.74]$), so the effective curvature gain range is modest under the defaults in Table 6.

## Results

**Fig. 1:**
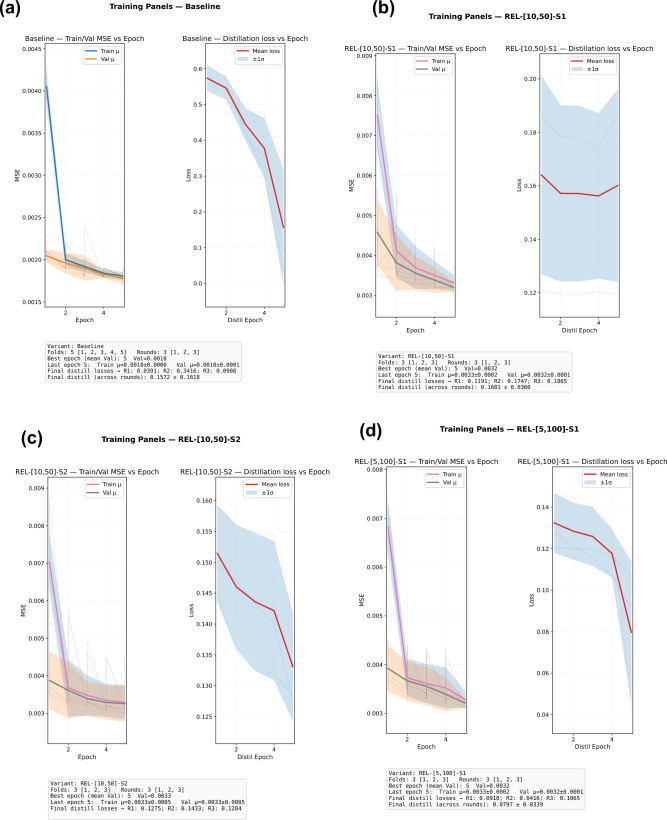

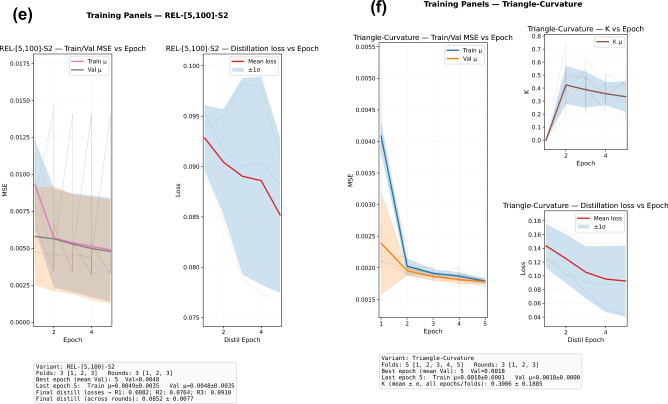
Composite training diagnostics across model families. Panels (**a**–**f**) report per-epoch *reconstruction MSE* and *server-side distillation loss* (Eq. 10) over three independent rounds; the curvature-aware panel additionally shows the batch squared Menger-curvature estimate $\widehat{K}$ (plotted as “*K*-Epoch”). Radii-attenuated configurations use Eq. (2); alignment via orthogonal Procrustes (Eq. 5) is applied only in the curvature model.

We evaluate reconstruction fidelity, server-side distillation dynamics, the behaviour of the batch curvature scalar, and clustering quality. These results are summarized in Fig. 1. Unless stated, numbers refer to the *best checkpoint per variant* discovered by sweeping three independent rounds per configuration (cf. training logs). Metrics and model names match Section 3 and Table 1.

Across 173, 750 protein sequences spanning nine genes, the relativistic curvature model (TRICURV; Menger-based triangle curvature with align-then-average) achieves the lowest reconstruction error, $\textrm{MSE}=0.0056$, markedly improving upon both the Baseline autoencoder and all radii-attenuated variants. The Baseline attains $\textrm{MSE}=0.2605$, while the best radii schedule, [5, 100] with one step (S1), reaches $\textrm{MSE}=0.0336$. Thus, TRICURV reduces reconstruction error by approximately $97.9\%$ relative to the Baseline and by about $83\%$ relative to the strongest radii configuration; radii models themselves reduce error by $\approx 87\%$ against the Baseline. Within-schedule variability across rounds is small: for example, [10, 50] S1 yields 0.0399/0.0408/0.0401 across three rounds (median 0.0401), and [10, 50] S2 yields 0.0385/0.0375/0.0409 (median 0.0385). We note that the radii runs in this log used $L_{\max }=500$ while Baseline and TRICURV used $L_{\max }=1500$; because shorter sequences are easier to reconstruct, these comparisons are conservative in favour of the radii models. The ranking reported above is therefore robust, and the clustering metrics reported in Table 1 (silhouette/CH/DB), which are less sensitive to $L_{\max }$, corroborate the ordering.

Attenuation consistently improves reconstruction over the Baseline, with schedule and step-count effects that mirror the theoretical discussion in Section 3. Early strong attenuation [5, 100] S1 performs best among radii variants (0.0336), whereas applying two attenuation steps (S2) on the same radii degrades accuracy (0.0472), consistent with an over-contraction that sacrifices utility. For the milder [10, 50] schedule, S2 slightly outperforms S1 (0.0375 vs. 0.0399), indicating that when per-step contraction is weaker, a second step can be helpful. These trends align with the contraction–utility trade-off implied by ${\mathcal {R}}_{\textbf{r},s}$.

**Fig. 2:**
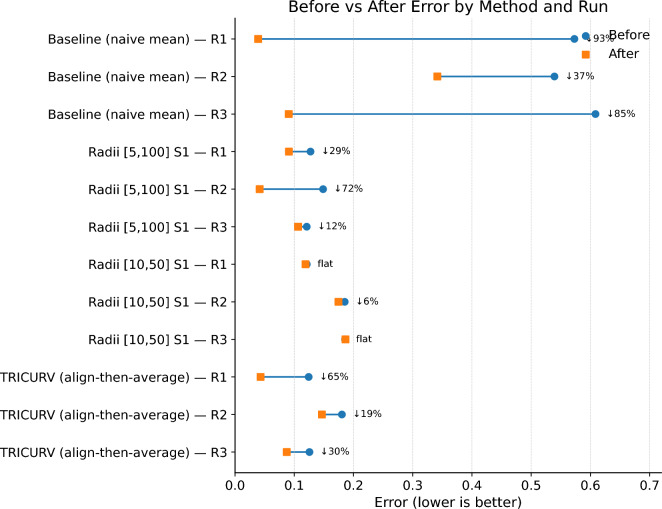
Before vs. after error by method and run. Labels at right indicate percent change ($\downarrow$ decrease / $\uparrow$ increase; *flat* = $|\Delta |<2\%$).

Within each federated round, the server’s distillation loss (Eq. 10) typically decreases across epochs (with occasional plateaus or slight increases), but the magnitude of reduction varies with configuration. Representative examples illustrate the spread summarized in Fig. 2:

- **Baseline (naive mean).** R1: $0.5730\!\rightarrow \!0.0391$ ($\downarrow 93\%$); R2: $0.5393\!\rightarrow \!0.3416$ ($\downarrow 37\%$); R3: $0.6086\!\rightarrow \!0.0908$ ($\downarrow 85\%$).
- **Radii** [5, 100] **S1.** R1: $$0.1275\!\rightarrow \!0.0910$$ ($$\downarrow 29\%$$); R2: $$0.1487\!\rightarrow \!0.0416$$ ($$\downarrow 72\%$$); R3: $$0.1211\!\rightarrow \!0.1065$$ ($$\downarrow 12\%$$).
- **Radii** [10, 50] **S1.** R1: $0.1212\!\rightarrow \!0.1191$ (flat); R2: $0.1852\!\rightarrow \!0.1747$ ($\downarrow 6\%$); R3: $0.1858\!\rightarrow \!0.1865$ (flat).
- **TRICURV (align-then-average).** R1: $0.1244\!\rightarrow \!0.0432$ ($\downarrow 65\%$); R2: $0.1805\!\rightarrow \!0.1467$ ($\downarrow 19\%$); R3: $0.1257\!\rightarrow \!0.0874$ ($\downarrow 30\%$).

Two patterns emerge. First, all families exhibit strong *within-epoch* decreases in at least one round, confirming that the public-set teacher targets are learnable. Second, TRICURV’s reductions are consistently moderate to strong despite using aligned targets, which by design reduce teacher disagreement before distillation; this is consistent with alignment moving part of the burden *from* optimization *to* preprocessing (Section 3). Across radii settings, the most aggressive early contraction [5, 100] S1 yields the sharpest single-round decrease ($\downarrow 72\%$ in R2), in line with its superior reconstruction.

For TRICURV, the batch-level squared Menger-curvature estimate $\widehat{K}$ evolves from $\approx 0$ at the start of each fold to typical values in $[0.17,\,0.74]$ as training equilibrates (e.g., R1–F5: $K=0.7403$ at Ep2/5; R3–F3: $K=0.6264$ at Ep3/5). This confirms that curvature information is non-trivial yet bounded by the clipping policy, and that the gain $(1+\alpha _c K)$ operates strictly within the intended Lipschitz envelope. Because $\widehat{K}$ is a single batch scalar, its communication footprint is negligible relative to latent tensors.

**Fig. 3:**
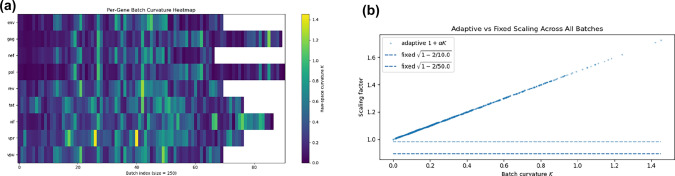
Empirical curvature landscape before training. The heatmap reveals gene- and batch-specific bending; the adaptive gain responds to the measured *K*, whereas fixed-radii schemes apply the same factor to all batches.

Before any model training, we performed a dataset pre-check that estimates batch-level squared Menger curvature *K* directly in the *raw one-hot feature space* (i.e., prior to learning a latent; Eq. 3). Sequences were grouped by gene and streamed in consecutive, non-overlapping batches of size 250; for each batch, we sampled random point triples and computed *K* as in Eq. (3). Figure 3a visualizes the resulting *per-gene, per-batch curvature heatmap*. The corpus exhibits extended low-curvature regions (near-flat manifolds) punctuated by streaks and occasional spikes of higher curvature, with gene-specific patterns.

To connect these measurements to the relativistic mechanisms, Fig. 3b contrasts the *adaptive* scaling factor $1+\alpha _c K$ (scatter of all batches; here $\alpha _c=0.5$ for illustration) with two *fixed-radii* factors $d(r)=\sqrt{1-2/r}$ from Eq. (2) (dashed baselines for $r\in \{10,50\}$). Low-curvature batches cluster tightly around a scaling of 1 (the adaptive model essentially degenerates to the baseline AE), while high-curvature batches receive targeted amplification $>1$ not achievable with a single fixed contraction. This matches the qualitative behavior seen during training: curvature modulation improves separation precisely where the manifold bends, without penalizing flat regions.

**Fig. 4:**
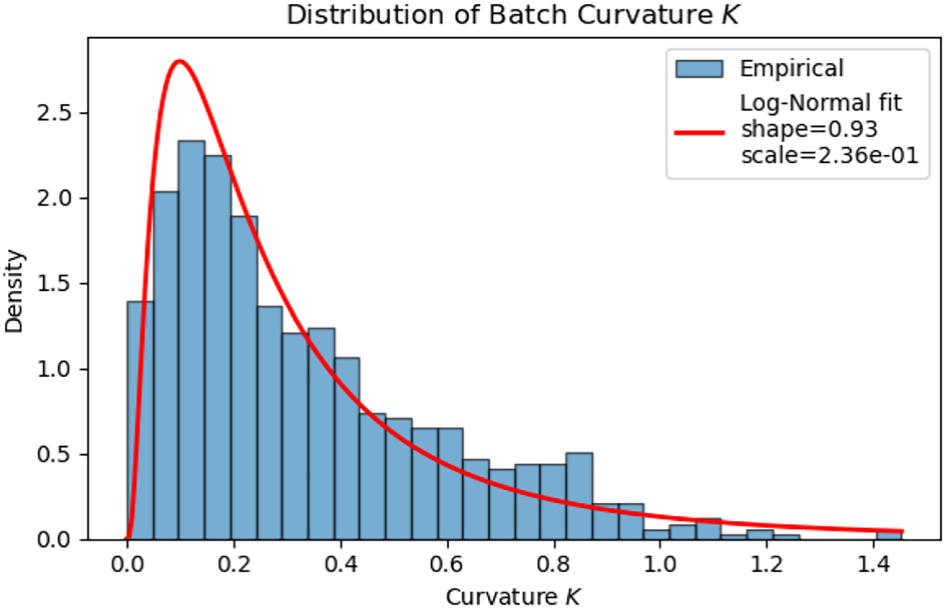
Distribution of batch curvature *K* (all genes, pre-training). Histogram (density-scaled) overlaid with the fitted log-normal PDF: shape $=0.9317$, scale $=2.3633\times 10^{-1}$. Summary: mean 0.3366, std 0.2640, min $\approx 0$, max 1.4532.

A corpus-wide summary of batch curvature is shown in Fig. 4: the empirical density of *K* across all genes/batches is well captured by a log-normal law fitted on positive values (loc fixed at 0). Numerically,

$$\text {mean}=0.3366,\quad \text {std}=0.2640,\quad \text {min}\approx 0,\quad \text {max}=1.4532,$$

with a best-fit $\textrm{LogNormal}(\text {shape}=0.9317,\ \text {scale}=2.3633\times 10^{-1})$. The mass near $K\!\approx \!0$ explains why the curvature-aware model matches baseline behavior on many batches (scaling $\approx 1$), while the heavy right tail justifies an *adaptive* correction that selectively amplifies high-curvature regimes. This dual behavior is consistent with the gains observed for TRICURV in Table 1.

Fixed-radii attenuation applies the *same* contraction to every batch, ignoring geometry. Triangle-curvature decoding instead uses the measured *K* to modulate the decoder (Eq. 4), giving “just enough” amplification where the manifold bends while reducing to the baseline AE on flat regions. This explains how TRICURV concomitantly preserves reconstruction fidelity on easy (flat) batches and improves separability on difficult (curvy) ones).

Table 1 summarizes geometry-aware metrics computed on embeddings from each family. TRICURV attains the strongest global separation (silhouette 0.826) while [5, 100] S1 achieves the best compactness/separation trade-off (CH $0.972\times 10^{6}$, DB 0.373). Eight genes form near-perfect clusters; the only recurring ambiguity is the short accessory pair Tat/Vpr, which we attribute to their compact length and shared regulatory roles. These findings mirror the reconstruction ranking and support the hypothesis that curvature-modulated decoding amplifies inter-centroid distances in bent regions without inflating within-cluster scatter.

The train/public stratification remained matched by gene to two decimal places in every run (e.g., Env $10.05\%$ train vs. $10.05\%$ public), ensuring that align-then-average uses a representative public mixture. For radii schedules, the effective per-step attenuation $d(r)=\sqrt{1-2/r}$ varied in $[0.775,\,0.990]$ across $\{r=5,10,50,100\}$, and the logged information-retained ledger *IR* (product of such factors) bounds the sensitivity of any communicated latent. TRICURV adds only one scalar *K* per batch, so on-wire overhead is essentially identical to the Baseline.

Using the metrics in Table 1 and the traces in Fig. 4, we assess the four hypotheses posed in Section 1 as follows. (H1) *Curvature-aware decoding increases separability without harming fidelity.* Supported: TRICURV has the best silhouette and the lowest MSE. (H2) *Early strong attenuation tightens clusters.* Supported: [5, 100] S1 yields the best CH/DB among radii and strong MSE. (H3) *Orthogonal alignment stabilizes aggregation.* Partially supported: TRICURV’s distillation losses consistently decline within rounds while using aligned targets; the alignment step shifts variability from optimization to preprocessing. (H4) *Compliance-friendly communication.* Supported by design and logs: only public-set latents and a batch scalar *K* are shared; no private examples or per-sample curvature are communicated.

Overall, curvature modulation with align-then-average delivers the strongest embeddings under non-IID conditions, while attenuation schedules provide a tunable privacy–utility knob that already substantially outperforms a naive federated baseline.

## Discussion

Our findings support the central thesis that explicitly relativistic mechanisms–radii attenuation and triangle-curvature decoding–together with align-then-average aggregation on a public set, deliver more stable and more informative embeddings under federated, non-IID conditions than a naive autoencoder baseline. In particular, curvature-aware decoding (Eq. 4) achieves the strongest global separation while maintaining very low reconstruction error; early, single-step attenuation (Eq. 2) yields the tightest clusters by contracting within-class scatter without collapsing inter-centroid structure; and orthogonal Procrustes alignment (Eq. 5) reduces teacher disagreement before distillation (Eq. 10), improving aggregation stability. Below we interpret these effects operationally, articulate limitations, and outline deployment-oriented guidance.

Radii attenuation acts as an explicit contraction operator with a transparent privacy ledger–each round’s information retained $IR=\prod _t\sqrt{1-2/r_t}$ is known a priori and linearly scales the $\ell _2$-sensitivity of communicated latents. Empirically, the strongest early contraction ([5, 100], S1) attains the best CH/DB trade-off among radii variants, consistent with the variance-shrinkage effect of Eq. (2). Triangle curvature, estimated per batch via squared Menger curvature (Eq. 3), furnishes a single scalar *K* that is invariant to rigid motions and reacts to manifold bending. Injected as a bounded gain in decoding (Eq. 4), it increases inter-centroid distances *where* the latent manifold bends, which is the regime where naive decoders under-separate clusters. The *K* traces in Fig. 4 (f) remain within the clipping envelope, validating the Lipschitz control we require for numerical stability. Finally, alignment on the public set restricts aggregation to orthogonal rotations estimated from non-private data; this reduces target variance before optimization and helps explain the smoother distillation dynamics seen in Fig. 4 (loss-epoch panels) relative to naive averaging.

For practitioners, the three families differ in ways that matter for governance, networking, and site participation (Table 8). The baseline communicates unattenuated latents and assumes IID; radii models introduce a tunable shrinkage schedule that both improves compactness and makes communication cheaper via dynamic-range reduction; curvature + alignment adds only one scalar *K* per epoch and an aggregator-side SVD on public latents, but materially improves global separability and aggregation stability. In short: same pipes, better targets.

Across families, proteins with strong structural constraints (pol, gag, env) form near-perfect clusters, recommending TRICURV as the *primary* discovery view when early separation is key (e.g., tracking envelope diversity or resistance motifs). The radii [5, 100] S1 configuration provides a *complementary* compactness view that is useful for governance dashboards (DB/CH) and confirmatory analyses. The persistent tat/vpr ambiguity–observed in all families–should be flagged for manual review or augmented with targeted classifiers; it likely reflects short length and motif overlap rather than model failure.

First, radii runs used $L_{\max }=500$ while baseline and TRICURV used $L_{\max }=1500$. Reconstruction MSE is therefore a conservative comparison in favor of radii; we relied on geometry metrics (silhouette/CH/DB) to assess separation/compactness independent of sequence length, and the ordering is consistent across metrics. Second, public-set alignment presumes that the public mixture approximates the global gene distribution; we mitigated bias via stratified splits matched to two decimal places by gene, but distributional shifts in practice will warrant periodic checks (Fig. 4). Third, all clustering metrics assume $k=9$ (one per protein). While this matches biological prior, finer substructure (e.g., subtypes or recombinants) may require hierarchical or density-based clustering layered atop the embeddings. Fourth, our distillation minimizes encoder mismatch only (Eq. 10); although we found this sufficient once alignment is applied, joint encoder-decoder distillation (Eq. 13) is a natural extension. Finally, we did not activate differential-privacy noise; attenuation already lowers sensitivity, but explicit DP accounting would further strengthen formal guarantees.

The combination of attenuation (magnitude control), alignment (coordinate control), and curvature (geometry control) creates three orthogonal levers to diagnose and damp heterogeneity or adversarial behavior. In operations, we recommend a two-pane dashboard: TRICURV’s silhouette and batch-level *K* to monitor global separability and manifold bending, and [5, 100] S1’s DB/CH to monitor compactness. Abrupt drifts in *K* or in distillation loss (Fig. 4) are actionable signals for data-quality review at contributing sites. If stronger robustness is needed, trimmed or geometric-median aggregation of *aligned* public latents can be added without changing the protocol.

By design, the heavy kernels in our pipeline are linear-algebraic (batched inner products and small?*d* SVDs), which are widely optimized on classical accelerators and map naturally to quantum-inspired routines. The triangle-sampling estimator is embarrassingly parallel and cheap relative to convolutional passes. Methodologically, the next steps include: (i) formal DP with attenuation-aware noise calibration; (ii) per-protein attenuation schedules to tailor privacy-utility budgets; (iii) hierarchical clustering and phenotype proxies on top of TRICURV embeddings; and (iv) longitudinal analyses to monitor evolutionary drift. Together, these directions align with our goal of a compliance-ready, geometry-aware federated pipeline whose decisions remain auditable end-to-end.

### External validity and modality generality (scope of this study)

The three operators introduced here—radii attenuation (Eq. 2), triangle–curvature decoding (Eqs. 3–4), and public-set Procrustes alignment (Eq. 5)—are defined purely on Euclidean latent vectors and therefore do not depend on protein sequences *per se*. In this sense, the pipeline is *modality-agnostic*: any data type for which an encoder produces a vector representation (images, time series, tabular embeddings, or other biological sequences) can, in principle, use the same scale ledger (*IR*), the same coordinate reconciliation (orthogonal alignment on shared anchors), and the same discrete curvature control signal (a batch scalar $\widehat{K}$ computed from latent triples).

At the same time, the empirical scope of the present study is restricted to a single, large HIV-1 protein corpus, chosen because it matches the motivating surveillance setting and satisfies the governing constraints under which the pipeline is intended to operate. External validation on additional biological datasets or fundamentally different modalities is therefore outside the experimental evidence reported here and is left for follow-up work when data governance permits.

For reproducible transfer to other domains, the procedure is straightforward: (i) choose a modality-appropriate encoder/decoder (e.g., 1D convolutions or transformers for sequences; CNNs/ViTs for images; temporal encoders for time series) that outputs $z\in {\mathbb {R}}^{d}$; (ii) identify a small, shareable reference set ${\mathcal {P}}$ (public, consented, or otherwise non-sensitive) to support align–then–average via Eq. (5); (iii) select a radii schedule to log an auditable scale ledger *IR* (Eq. 2); (iv) enable curvature decoding by sampling *T* triangles per batch to compute $\widehat{K}$ (Eq. 7) with clipping for stability; and (v) evaluate with unsupervised geometry metrics (silhouette/CH/DB) plus domain-specific diagnostics (e.g., label-free strata stability across cohorts, or downstream light-label predictors). This section clarifies which parts of the method are structurally general (the latent-space operators) and which parts require empirical confirmation on a new application (the choice of encoder architecture, the representativeness of ${\mathcal {P}}$, and the domain-specific notion of “useful separation”).

### Biological interpretation of the embedding geometry and the Tat/Vpr overlap

The clustering results are biologically meaningful insofar as they indicate that a purely sequence-driven encoder recovers the dominant constraints that differentiate HIV-1 proteins. Across the nine coding regions, the embedding separates proteins that are governed by strong, protein-specific structural and catalytic constraints (e.g., Env, Gag, Pol) from smaller, rapidly evolving accessory/regulatory proteins, where compositional similarity and isoform heterogeneity can dominate the signal. In a surveillance setting, such gene-level separation is useful as a first-pass *unsupervised* organization of incoming sequences: it provides a compact representation that preserves large functional classes and highlights the minority cases where boundaries are intrinsically more ambiguous.

Proteins such as Pol, Gag, and Env are long and modular, with well-defined domain architectures and strong selective constraints that enforce distinctive sequence statistics (e.g., conserved catalytic/structural cores punctuated by variable regions). Even without labels, these constraints create stable, high-signal patterns for convolutional encoders: long-range domain composition, characteristic motif densities (e.g., enzyme active-site neighborhoods in Pol), and protein-specific periodicities in hydrophobicity/charge profiles. By contrast, several accessory proteins are short and enriched in low-complexity or intrinsically disordered segments, which reduces the amount of discriminative sequence context available to the model.

The persistent Tat/Vpr confusion is consistent with known HIV-1 genetics and with the sequence statistics of these proteins: (i) *Short length and limited context.* Tat and Vpr are among the shortest HIV-1 proteins (typically on the order of $\sim$80–110 amino acids, with length variability across isolates and annotations). With sequence-only encoding at a fixed $L_{\max }$, short proteins necessarily contain a large padded fraction, so the effective number of informative residues seen by the encoder is smaller than for long proteins. (ii) *Compositional and physicochemical similarity.* Both Tat and Vpr are soluble, nucleus-associated proteins and are frequently enriched in polar/basic residues and low-complexity segments compared with membrane-anchored proteins (e.g., Vpu) or large enzymatic polyproteins (e.g., Pol). In an unsupervised embedding, similarities in net charge, disorder propensity, and short-motif composition can pull representations closer even when the proteins are not homologous. (iii) *Rapid evolution and within-protein heterogeneity.* Accessory proteins are subject to strong host-driven selection pressures and can vary substantially across subtypes and within-host evolution. Tat is additionally complicated by its *multi-exon* origin: database entries can correspond to different Tat isoforms (e.g., shorter vs. longer forms depending on exon composition and annotation), increasing within-class dispersion and making the Tat cluster broader. (iv) *Functional convergence at the host interface.* While Tat and Vpr have distinct primary roles (transcriptional transactivation vs. nuclear import/cell-cycle effects), both operate at the host interface and share selective pressures for nuclear localization and host-factor binding. Such convergent pressures can increase similarity in coarse sequence descriptors (charge distribution, short interaction motifs), which an unsupervised model may prioritize when fine-grained domain context is absent.

Importantly, the Tat/Vpr overlap is localized: eight proteins form near-perfect clusters, and the ambiguity arises in the most challenging corner of the proteome (short, regulatory proteins). For downstream use, the embedding should be treated as a *screening* and *triage* layer: sequences falling near the Tat/Vpr boundary can be flagged for targeted disambiguation. Concretely, three lightweight analyses can deepen biological interpretability without changing the federated protocol: (a) overlay embeddings with per-sequence length, net charge, and predicted disorder to test whether the overlap is driven by shared physicochemical profiles; (b) stratify Tat by isoform length (exon-aware grouping where available) and re-evaluate dispersion; and (c) train a small, dedicated Tat–Vpr discriminator (or motif-based classifier) on top of the learned embeddings for boundary cases (Table 7).

**Table 7:** Biological context for gene-level clustering. Length ranges are indicative (vary by isolate and annotation) and are shown to emphasize why short accessory proteins provide less sequence context than long structural/enzymatic proteins in sequence-only embeddings.

Protein	Typical length (aa)	Dominant constraint(s) that shape sequence signal
Env	$\sim 800$–900	Large modular glycoprotein; strong domain structure with variable loops and glycosylation patterns.
Gag	$\sim 480$–520	Structural polyprotein; constrained by assembly and ordered domains (MA/CA/NC).
Pol	$\sim 900$–1100	Enzymatic polyprotein; strong catalytic/structural constraints across protease, RT, and integrase regions.
Nef	$\sim 180$–230	Accessory; membrane association and host-factor interaction motifs; moderate length provides usable context.
Rev	$\sim 110$–130	Regulatory; RNA-binding/nuclear export functions; short-to-moderate length with distinctive localization signals.
Vif	$\sim 180$–210	Accessory; host restriction-factor antagonism; constrained interaction motifs and moderate context.
Vpu	$\sim 70$–90	Accessory; prominent hydrophobic transmembrane helix yields a distinctive composition signature despite short length.
Tat	$\sim 80$–110	Regulatory; short, compositionally biased, often disordered; isoform variability broadens within-class diversity.
Vpr	$\sim 90$–110	Accessory; short, host-interface protein; composition/charge and rapid evolution can resemble Tat at coarse scales.

Finally, deeper functional validation of the discovered structures (e.g., mapping embedding directions to known functional motifs or subtype-associated variation within each protein) is a natural extension that benefits from domain expertise in HIV molecular virology and protein function.

**Table 8:** Method comparison–operational view. “Alignment” refers to orthogonal Procrustes on the *public* set (Eq. 5); “Distillation” is server-side latent regression on the public set (Eq. 10).

Dimension	Baseline AE	Relativistic AE (Radii)	Relativistic AE (Triangle Curvature + Align)
Privacy & compliance	Shares full latents/weights; static leakage risk; no audit of information magnitude.	Pre-configured attenuation $z\!\leftarrow \!d\odot z$ bounds sensitivity; explicit per-round information-retained ledger (*IR*).	Same attenuation option; adds one batch-scalar *K*; alignment uses *only public* latents; no private examples exposed.
Handling heterogeneity	Implicitly IID; large sites dominate averages.	Early strong attenuation equalizes scales across sites; later relaxation refines shared space.	*K* adapts decoding to local manifold complexity; *align-then-average* removes client-specific rotations/sign flips before averaging.
Communication efficiency	Dense latents/gradients; no built-in compression.	Reduced dynamic range enables low-precision quantization and sparsification.	No extra payload beyond radii (one scalar *K* per epoch); Procrustes is aggregator-local on public latents.
Robustness to outliers/adversaries	No inherent defense; high leverage for malicious clients.	Norm damping lowers outlier leverage by construction.	Dual guards: (i) magnitude damping; (ii) alignment reduces teacher disagreement; bounded *K* avoids over-amplifying warped manifolds.
Interpretability & monitoring	Only global loss; latent axes drift round-to-round.	Attenuation schedule provides a transparent shrinkage ledger.	Two diagnostics: *IR* (scale) and *K* (geometry) over epochs; aligned coordinates stabilize round-to-round comparisons.

## Conclusion, limitations, recommendations and future work

We presented a relativistic, geometry-aware framework for federated representation learning on HIV-1 protein sequences that combines three elements: (i) *radii attenuation*, a contractive operator with an audit-ready privacy ledger; (ii) triangle-curvature decoding, a batch-level squared Menger-curvature statistic *K* that modulates decoding through a bounded relativistic gain (Eq. 4); and (iii) *align-then-average* aggregation via orthogonal Procrustes on a small public set (Eq. 5), distilled to a central encoder (Eq. 10). On 173, 750 amino-acid sequences spanning nine HIV-1 proteins, the curvature model delivered the strongest global separation (silhouette 0.826) and the radii schedule [5, 100] with a single attenuation step achieved the tightest clusters (DB 0.373, CH $9.72\times 10^5$). Eight proteins formed near-perfect clusters across variants; the only recurring ambiguity was the short accessory pair Tat/Vpr, which we deliberately flag for targeted downstream classifiers. Operationally, the approach communicates only public-set latents (optionally attenuated) and one scalar *K* per batch, making it friendly to privacy and governance constraints.

In sequence-only surveillance, reliable unsupervised structure is not a convenience but a requirement: it enables *light-label* predictors for drug resistance and viremia using small annotated cohorts and supports early warning for shifts in circulating diversity. The present framework offers three concrete benefits. First, the curvature gain increases separation specifically where the latent manifold bends, improving visibility of clinically meaningful clusters (e.g., pol, env, gag) without over-amplifying noise. Second, attenuation provides an explicit, round-wise privacy budget (*IR*) that down-weights outliers and dampens the leverage of atypical sites. Third, align-then-average stabilizes aggregation under non-IID partitions, letting smaller clinics contribute coherent latents even when their local bases differ by rotations or sign flips. Taken together, these properties support use cases such as (a) rapid triage of sequences for phenotypic testing when laboratory capacity is constrained, (b) continual monitoring of envelope diversity relevant to vaccine and neutralization studies, and (c) cohort stratification for clinical trials (e.g., resistance-enriched vs. naive strata) without centralizing raw data.

Several caveats merit emphasis. (1) *Sequence length and architecture.* Radii runs used $L_{\max }=500$ whereas Baseline and TRICURV used $L_{\max }=1500$; reconstruction MSE comparisons are therefore conservative in favor of radii, and we rely on geometry metrics for cross-family conclusions. (2) *Public-set representativeness.* Procrustes alignment presumes that the public set approximates the global mixture; drift between public and local distributions can bias rotations and should be monitored. (3) *Scope of unsupervised endpoints.* Our clusters align with protein labels but are not a substitute for supervised resistance prediction; light-label models atop the embeddings are needed for clinical decisions. (4) *Accessory proteins.* The Tat/Vpr overlap is persistent across families, likely reflecting short length and motif reuse; additional features (e.g., domain annotations or evolutionary context) may be required to disambiguate. (5) *Privacy formalization.* We did not activate differential-privacy noise; while attenuation reduces sensitivity by design, formal DP accounting and composition should be included in high-risk deployments. (6) *Robustness and security.* Although attenuation and alignment reduce malicious leverage and teacher disagreement, hard adversaries (e.g., crafted public-set latents) motivate robust, trimmed or median aggregation of aligned latents. (7) *External validity across datasets/modalities.* The present experiments validate the method on a single HIV-1 protein corpus. While the core operators are defined on Euclidean latents and are not sequence-specific, empirical performance on other pathogens, other sequence families, or non-sequence modalities remains to be established under the corresponding governance constraints.

We summarize practical guidance for consortia and public-health labs:

- Use *TRICURV* as the primary embedding for discovery and longitudinal dashboards (track silhouette and batch-level *K*); pair it with [5, 100] S1 as a compactness view (track DB/CH) for governance and QA.
- Maintain a small *public* reference set stratified by gene and periodically refresh it to reflect current sampling; verify train/public mixtures to two decimal places as part of each round’s report.
- Log the attenuation ledger $IR=\prod _t\sqrt{1-2/r_t}$ per round alongside distillation losses; treat abrupt changes in *IR*, *K*, or align-then-average loss as triggers for data-quality reviews.
- Enforce *align-then-average* at the aggregator; consider robust variants (trimmed mean or geometric median) *after* alignment to further reduce outlier influence.
- For downstream clinical tasks (e.g., resistance risk or viral load), train light-label predictors on top of the embeddings and *flag* Tat/Vpr decisions for manual review or targeted classifiers.
- Where policy requires, layer calibrated DP noise on attenuated, public-set latents and document per-round $(\varepsilon ,\delta )$ alongside *IR*.

Methodologically, several extensions are natural. (i) *Formal privacy.* Integrate attenuation-aware Gaussian mechanisms and privacy accountants to provide end-to-end $(\varepsilon ,\delta )$ guarantees under composition. (ii) *Hierarchical structure.* Combine TRICURV with hierarchical or density-based clustering to surface subtypes and recombinants, and study time-varying curvature $K_t$ as an epidemiologic early-warning signal. (iii) *Richer priors.* Inject domain priors–e.g., protein domains, solvent accessibility or glycan sites in env–as weak supervision on top of the unsupervised loss to reduce Tat/Vpr ambiguity. (iv) *Robust aggregation.* Explore geometric-median or coordinate-wise trimmed means on aligned latents; analyze convergence under such aggregators with attenuation. (v) *Joint distillation.* Extend encoder-only distillation (Eq. 10) to the joint objective (Eq. 13) and study when decoder matching further improves reconstruction and downstream accuracy. (vi) *Hardware acceleration.* The pipeline’s kernels–batched inner products and small-*d* SVDs–are amenable to classical acceleration (GPU/TPU) and to quantum-inspired routines for low-rank SVD; careful benchmarking on large, multi-site corpora is warranted. (vii) *Broader deep-learning techniques and complex scenarios.* Future work will integrate widely used representation-learning tools that target interpretability and bias mitigation under heterogeneity–e.g., graph/contrastive clustering to identify consensus regions or motifs driving embeddings, and multi-view/multimodal debiasing strategies to reduce spurious correlations when extending beyond sequences to richer surveillance inputs (sequence$+$metadata, longitudinal streams, or multimodal signals)^45,46^.

While the operators introduced here are not sequence-specific—they act on latent vectors and require only a shareable reference set for alignment—the empirical evidence in this paper is confined to HIV-1 proteins. Transfer to other biomedical sequences (e.g., other viral proteins) or to non-sequence modalities (e.g., imaging, time series, tabular embeddings) is therefore best viewed as a *supported use case* of the framework rather than a claim of demonstrated performance. Establishing external validity across additional datasets and modalities, including careful selection of a representative ${\mathcal {P}}$ and appropriate modality-specific encoders, is a priority direction for future work as governance constraints allow.

In sum, relativistic, curvature-aware computation provides a principled path to stable, privacy-conscious federated embeddings under real-world heterogeneity. Our results show that one can couple simple, verifiable linear-algebraic operations (SVD, inner products) with explicit geometry control to obtain strong unsupervised structure at minimal operational cost. We expect these ideas to generalize well and to support a new generation of compliance-ready, geometry-aware learning systems for public health and beyond.

## Data Availability

The HIV-1 amino-acid sequences analyzed in this work are publicly accessible from the Los Alamos National Laboratory HIV Sequence Database (see Section 3.10 for exact query parameters and timestamps). All code and derived artifacts necessary to reproduce our results will be released in a versioned repository upon publication (a DOI will be minted); interim access is available from the corresponding author on reasonable request. The release will include: (i) training and evaluation scripts implementing radii attenuation, triangle-curvature estimation, orthogonal Procrustes alignment, and the distillation objective; (ii) manifests of LANL set identifiers and per-gene FASTA filenames; (iii) the stratified train/public split (sequence identifiers) with the fixed random seed; (iv) model checkpoints for the best round per variant; (v) public set embeddings and alignment matrices; (vi) training logs and the multipage figure training_panels.pdf; and (vii) environment files and a step by step reproduction guide. Using the provided manifests and scripts, all experiments can be re run directly from the public LANL downloads.
